# Supplementary material for: Impact of heavy precipitation events on pathogen occurrence in estuarine areas of the Puzi River in Taiwan
Source: PLoS One. 2021 Aug 16;16(8):e0256266. doi: 10.1371/journal.pone.0256266 (PMC8366992; doi:10.1371/journal.pone.0256266)
Supplement: S1 Table — (DOCX) [file pone.0256266.s001.docx]

**S1 Table. Water quality parameters variation of Puzi River (site A-B) after rainfall.**

| Sampling events | Days after heavy precipitation (by East Asian Rainy) | | | | Days after extreme heavy precipitation  (by Typhoon Nepartak) | | | |
| --- | --- | --- | --- | --- | --- | --- | --- | --- |
| Water quality parameters | Day 1 | Day 3 | Day 8 | Day 12 | Day 1 | Day 3 | Day 8 | Day 12 |
| Heterotrophic plate count (CFU/mL) | 48,067±30,067 | 66,950±37,183 | 953±133 | 1,394±880 | 221,133±188,333 | 948,700±821,900 | 94,866±18,861 | 57,483±12,938 |
| Total Coliform (CFU/100mL) | 16,638±1,356 | 7,358±386 | 3,680±1,284 | 273±94 | 4,068±5,538 | 2,307±1,290 | 1,928±433 | 101±68 |
| *Escherichia coli* (CFU/100mL) | 1,045±307 | 292±4 | 95±57 | 2±2 | 450±336 | 116±104 | 334±150 | 37±20 |
| pH | 7.54±0.04 | 7.67±0.05 | 7.46±0.06 | 7.86±0.04 | 8.18±0.28 | 7.70±0.07 | 7.78±0.17 | 7.63±0.17 |
| Turbidity | 140.80±63.20 | 49.30±19.80 | 54.43±10.62 | 2.84±0.70 | 119.35±54.65 | 17.74±4.85 | 67.00±27.00 | 19.90±5.66 |
| Salinity (%) | 0.28±0.08 | 0.99±0.90 | 0.38±0.38 | 0.95±0.91 | 0.48±0.18 | 0.04±0.24 | 0.18±0.23 | 0.37±0.09 |
| Dissolved oxygen (mg/L) | 5.11±0.01 | 5.04±0.09 | 4.11±0.11 | 3.79±0.10 | 7.51±0.01 | 4.50±0.03 | 3.30±0.04 | 4.03±0.45 |
| Average water temperature (°C) | 26.03±0.10 | 28.05±0.19 | 28.87±0.53 | 29.03±0.19 | 26.05±0.10 | 27.20±0.43 | 29.98±0.12 | 30.03±0.05 |
