# Supplementary material for: Impact of heavy precipitation events on pathogen occurrence in estuarine areas of the Puzi River in Taiwan
Source: PLoS One. 2021 Aug 16;16(8):e0256266. doi: 10.1371/journal.pone.0256266 (PMC8366992; doi:10.1371/journal.pone.0256266)
Supplement: S2 Table — (DOCX) [file pone.0256266.s002.docx]

**S2 Table. Water quality parameters variation of Dongshing Fishing Port (site C-E) after rainfall.**

| **Sampling events** | **Days after heavy precipitation (by East Asian Rainy)** | | | | **Days after extreme heavy precipitation  (by Typhoon Nepartak)** | | | |
| --- | --- | --- | --- | --- | --- | --- | --- | --- |
| **Water quality parameters** | **Day 1** | **Day 3** | **Day 8** | **Day 12** | **Day 1** | **Day 3** | **Day 8** | **Day 12** |
| **Heterotrophic plate count (CFU/mL)** | **111,444±17,674** | **55,278±19,181** | **10,268±2,933** | **5,273±4,390** | **385,900±101,440** | **163,200±66,087** | **14,354±17,991** | **9,200±2,393** |
| **Total Coliform (CFU/100mL)** | **5,075±1,316** | **5,005±2,151** | **430±222** | **25±8** | **2,417±1,103** | **1,049±543** | **386±98** | **28±11** |
| ***Escherichia coli* (CFU/100mL)** | **232±25** | **467±145** | **55±11** | **2±1** | **28±5** | **95±36** | **124±49** | **24±21** |
| **pH** | **7.64±0.13** | **7.45±0.03** | **7.93±0.14** | **7.97±0.07** | **8.05±0.14** | **7.78±0.06** | **7.90±0.06** | **7.96±0.02** |
| **Turbidity** | **36.97±2.96** | **21.67±4.43** | **11.43±4.15** | **2.11±0.54** | **24.28±2.05** | **12.54±4.85** | **10.64±1.94** | **8.89±2.53** |
| **Salinity (%)** | **4.65±0.08** | **11.03±0.69** | **15.18±1.46** | **19.90±1.76** | **2.16±0.60** | **1.02±1.18** | **6.63±5.69** | **18.23±0.43** |
| **Dissolved oxygen (mg/L)** | **5.43±0.14** | **4.81±0.11** | **4.49±0.08** | **4.32±0.23** | **8.89±0.97** | **4.79±0.26** | **3.35±0.79** | **5.82±0.31** |
| **Average water temperature (°C)** | **26.65±0.02** | **27.01±0.20** | **28.71±0.16** | **29.07±0.48** | **26.08±0.04** | **28.09±0.21** | **29.10±0.26** | **29.77±0.24** |
