# Supplementary material for: Impact of heavy precipitation events on pathogen occurrence in estuarine areas of the Puzi River in Taiwan
Source: PLoS One. 2021 Aug 16;16(8):e0256266. doi: 10.1371/journal.pone.0256266 (PMC8366992; doi:10.1371/journal.pone.0256266)
Supplement: S3 Table — (DOCX) [file pone.0256266.s003.docx]

**S3 Table. Water quality parameters variation of oyster farms (site F-H) after rainfall.**

| Sampling events | Days after heavy precipitation (by East Asian Rainy) | | | | Days after extreme heavy precipitation  (by Typhoon Nepartak) | | | |
| --- | --- | --- | --- | --- | --- | --- | --- | --- |
| Water quality parameters | Day 1 | Day 3 | Day 8 | Day 12 | Day 1 | Day 3 | Day 8 | Day 12 |
| Heterotrophic plate count (CFU/mL) | - | 68,467±26,017 | 32,739±17,049 | 2,806±2,195 | 305,027±166,087 | 1,116,200±995,865 | 26,167±71,941 | 25,499±9,322 |
| Total Coliform (CFU/100mL) | - | 4,359±3,826 | 1±1 | 1±1 | 1,244±1,030 | 477±394 | 11±8 | 43±3 |
| *Escherichia coli* (CFU/100mL) | - | 13±6 | 8±3 | 0 | 27±85 | 44±19 | 0 | 16±7 |
| pH | - | 7.70±0.08 | 7.63±0.15 | 7.85±0.11 | 8.10±0.06 | 7.62±0.05 | 7.98±0.02 | 7.75±0.11 |
| Turbidity | - | 14.26±2.31 | 6.93±4.69 | 0.80±0.05 | 43.62±25.89 | 57.58±37.15 | 49.23±2.12 | 9.57±5.61 |
| Salinity (%) | - | 10.98±3.97 | 16.03±2.53 | 21.34±1.61 | 5.25±2.04 | 1.88±0.09 | 18.05±6.31 | 22.32±0.68 |
| Dissolved oxygen (mg/L) | - | 4.36±0.14 | 3.97±0.16 | 3.29±0.17 | 5.81±0.36 | 4.21±0.37 | 6.45±0.25 | 4.29±0.44 |
| Average water temperature (°C) | - | 29.02±0.06 | 29.11±0.14 | 29.75±0.21 | 26.11±0.03 | 26.42±0.15 | 27.98±0.39 | 28.08±0.21 |
