# Supplementary material for: Impact of heavy precipitation events on pathogen occurrence in estuarine areas of the Puzi River in Taiwan
Source: PLoS One. 2021 Aug 16;16(8):e0256266. doi: 10.1371/journal.pone.0256266 (PMC8366992; doi:10.1371/journal.pone.0256266)
Supplement: Attachment — Submitted filename: Plos_one_Reviewer_comments_R4.docx [file pone.0256266.s004.docx]

Reviewer #1: Manuscript No.: PONE-D-20-34257

Title: Impact of heavy precipitation on pathogen occurrence in estuary with oyster farming

Authors: YI-JIA SHIH et al

Overview: The paper investigates the impact of heavy rainfall events on the bacterial and viral load in an estuary. The authors sampled at 8 different points in the estuary. Seasonal samples were collected on a roughly 3 month cycle presumably to provide a baseline. Additional sampling was conducted after a single heavy rainfall event (>80mm/24 hours) and a single very heavy rainfall event (>200mm/24 hours). Water samples were screened for basic chemistry and pathogen load, both bacterial and viral. The authors report variations in pathogenic Vibrio sp., and both adenovirus and norovirus.

General comments: While the study is interesting there appear to be a number of flaws in the experimental design. The data presented is an average of 8 relatively dispersed sample sites. There is no attempt to show how each of the sites varies. Since some of the sites are a kilometer up the estuary while others are offshore, there may be substantial variation in water chemistry and pathogen load that is not being reported. There is no discussion of the hydrography/oceanography of the sample area. Some of the errors indicate substantial variability on the sample data.

Response:

We thank the reviewer for this comment. We have added the variation in average water quality parameters of each sampling site in the supplementary material. Moreover, we have added the environmental characteristics of the Puzi River basin in the discussion section. The relevant sentence has been rewritten as follows: As inshore farming of oysters around the Puzi River estuary is the primary source of oysters in Taiwan, and human activities and livestock farms are located around around the Puzi River basin [48], the wastewater from the river is an important contributor to the water quality of the estuary. Therefore it is necessary to monitor the dynamics of microbial communities in these areas.

Comment:

Title: The title is misleading. The inclusion of oyster farming might suggest that the authors investigated pathogen load in oysters, which they did not. In fact, the relevance to oyster farming is somewhat tenuous. I strongly recommend the reference to an oyster farming region is removed.

Response:

We thank the reviewer for this comment. We have renamed the manuscript as follows: “Impact of heavy precipitation events on pathogen occurrence in estuarine areas of the Puzi River in Taiwan.””

Comment:

Abstract: A little long, but relevant.

Response:

We have rephrased the Abstract as suggested.

Comment:

Introduction: Appropriate The references to the previous work (bibliography number 27 and 28) of the authors on line 67-69 should be cited with this sentence.

Response:

We have rephrased the sentence on lines 67-69 as followed ”Recently, our group has demonstrated that seasonal changes affect the detection rates and concentrations of human adenovirus in aquatic environments, which is associated with human gastroenteritis. Evidence from previous studies also suggest that the physical determinants and chemical compositions of water bodies might vary seasonally and, consequently, affect microbial communities [27, 28].”

Comment:

Methods: The methodology is somewhat confusing. There are a number of issues that need to be clarified.

1. The locations of the sampling points is somewhat confusing in relation to the data collected. The first section indicates samples were collected adjacent to oyster farms. But no further information is given. Which samples? Where are the oyster farms?

Response:

We thank the reviewer for this suggestion. We have added the description in the Figure 1 caption and illustrated the different types of sampling sites as follows: “Figure 1. Sampling locations around the estuarine areas of the Puzi River in Taiwan Sites A to B were downstream of the Puzi River, sites C to E were fishing ports, and sites F to H were offshore oyster farms. The geographical coordinates (latitude , longitude) are as follows: A (23.458433, 120.177433), B (23.445306, 120.165667), C (23.452656, 120.138769), D (23.450560, 120.139850), E (23.451032, 120.137538), F (23.473775, 120.120292), G (23.444178, 120.132592), and H (23.410706, 120.120975).”

Comment:

2. The first section indicates that ‘seasonal’ samples were collected quarterly. However this is untrue according to the presented data. Seasonal samples were collected in February, May, July and November. This is not quarterly.

Response:

Sampling occurred once per season in this study. We also defined December to February as the winter season. We have added this description as follows: “Moreover, sample collection was also carried out quarterly once per season from winter (December to February was defined as the winter season) in 2016 to winter in 2017 to monitor seasonal changes in water quality background.”

Comment:

3. The water quality data and pathogen load data seems to be a mean of all 8 sample points. These points are reasonably distributed both in estuary and the surrounding coast. Some of the sample point appear to be 2 or 3 kilometers offshore. Is it reasonable to average all these points? Some of the data points have large error.

Response:

In this study, we would like to understand the variation in overall water quality parameters, and these two sampling sites were located downstream of the Puzi River near the estuary; thus, we analyzed them together. However, we have also added the variation in average water quality parameter variation at different sampling sites in the supplementary material.

Comment:

4. Day 1 sampling for the ‘heavy rain’ even is an average of only 5 samples. There is no explanation or indication which sites were excluded.

Response:

Only five samples were collected downstream of the Puzi River and at fishing port sites after the first heavy rain event because of limited sampling from the oyster farm sites. This paragraph has been rewritten as follows:

A total of eight sites were investigated during periods of precipitation events; however, only five samples were collected during rain events. The collection of three offshore samples were banned by the Taiwan Coast Guard Administration for safety reasons. Moreover, sample collection was also carried out quarterly once per season from winter (December to February was defined as the winter season) in 2016 to winter in 2017 to monitor seasonal changes in water quality background. The longitude and latitude for each sampling site are illustrated in Fig 1.

Comment:

5. References listed in table 2 and not in the same format as the rest of the manuscript and are NOT listed in the bibliography.

Response:

We have corrected the format and added the missing reference to the bibliography.

Comment:

6. I would expect the seasonal sampling to have run for a full 12 months, or more, to ensure that a complete seasonal cycle is evaluated. That would mean that 5 sample sets should have been collected, including a February set in year 2.

Response:

We have added the water quality parameters in February 2017 in Table 1. The data as follows:

| Water quality parameters | Sampling dates | | | | |
| --- | --- | --- | --- | --- | --- |
|  | 2016/2/24 (n=8) | 2016/5/13 (n=8) | 2016/7/18  (n=8) | 2016/11/18  (n=8) | 2017/2/8  (n=8) |
| Heterotrophic plate count (CFU/mL) | 354±1,387 | 12,366±5,218 | 38,912±11,310 | 3,566±1,623 | 335±1421 |
| Total Coliform (CFU/100mL) | 254±511 | 409±502 | 631±2,023 | 280±266 | 224±483 |
| *Escherichia coli* (CFU/100mL) | 23±79 | 48±40 | 130±255 | 20±22 | 22±83 |
| pH | 8.6±0.6 | 8.0±0.2 | 7.9±0.1 | 7.6±0.2 | 8.4±0.3 |
| Turbidity (NTU) | 15.2±20.8 | 11.9±28.2 | 30.2±34.6 | 27.3±27.1 | 16.7±19.8 |
| Salinity (%_o_) | 29.9±1.5 | 27.5±1.9 | 9.3±6.8 | 21.6±12.2 | 20.7±5.3 |
| Dissolved oxygen (mg/L) | 7.0±0.4 | 4.8±0.4 | 4.5±0.9 | 5.7±0.8 | 6.9±0.3 |
| Average water temperature (°C) | 18.2±0.9 | 29.9±0.7 | 28.9±0.4 | 27.0±1.2 | 20.2±1.2 |

Comment:

Results: Reasonably appropriate. A number of the tables and figures need to have more descriptive titles and legends. For instance in table 3, both *Vibrio parahaemolyticus* and adenovirus have an ‘asterisk’ but the reason is not explained. Figure 2 leaves the reader to figure out which scale (left or right) reads for the temperature and which for the rainfall. Several parameters in table 5 have an ‘asterisk’, probably to indicate that they are significant, but this is not made clear. Errors are provided in table 1 but not for the comparable data in table 4. This means the reader cannot judge the variability in the provided data. In table 1 a particular sample is indicated either positive or negative for a given parameter, no indication which sample is positive and which negative.

Response:

We have added the explanations of the meaning o ‘asterisks’ in the tables, and have added the stranded error in Table 4; the modified values are as follows:

Table 4. Water quality parameters around the estuarine areas of the Puzi River after heavy precipitation

| Sampling events | Days after heavy precipitation  (by East Asian Rainy) | | | |  |
| --- | --- | --- | --- | --- | --- |
| Water quality parameters | Day 1 (n=5) | Day 3 (n=8) | Day 8 (n=8) | Day 12 (n=8) | |
| Heterotrophic plate count (CFU/mL) | 86,093±20,618 | 63,142±12,910 | 16,366±7,819 | 3,378±1,720 | |
| Total Coliform (CFU/100mL) | 9,700±2,953 | 5,351±1,507 | 941±645 | 78±75 | |
| *Escherichia coli* (CFU/100mL) | 557±222 | 253±106 | 188±19 | 1±1 | |
| pH | 7.6±0.1 | 7.6±0 | 7.7±0.1 | 7.9±0 | |
| Turbidity (NTU) | 78.5±32.4 | 25.8±6.7 | 20.5±11.6 | 1.8±0.4 | |
| Salinity (%) | 2.9±1.1 | 8.5±2.1 | 11.8±2.7 | 15.7±2.2 | |
| Dissolved oxygen (mg/L) | 5.3±0.1 | 4.7±0.1 | 4.2±0.1 | 3.8±0.2 | |
| Average water temperature (°C) | 26.4±0.1 | 28.0±0.2 | 28.9±0.3 | 29.3±3.1 | |
| Cumulative precipitation (mm) | 161 | 165 | 167 | 169 | |
| Average daily precipitation (mm) | 53.7 | 33.0 | 16.7 | 12.1 | |

Table 5. Water quality parameters around the estuarine areas of the Puzi River after extreme heavy precipitation

| Sampling events | Days after extreme heavy precipitation  (by Typhoon Nepartak) | | | |  |
| --- | --- | --- | --- | --- | --- |
| Water quality parameters | Day 1 (n=8) | Day 3 (n=8) | Day 8 (n=8) | Day 12 (n=8) | |
| Heterotrophic plate count (CFU/mL) | 314,381±180,326 | 716,950±471,663 | 38,912±41,879 | 27,383±22,439 | |
| Total Coliform (CFU/100mL) | 2,390±1,639 | 1,149±823 | 631±708 | 52±87 | |
| *Escherichia coli* (CFU/100mL) | 133±128 | 81±33 | 130±95 | 24±21 | |
| pH | 8.1±0.1 | 7.7±0.1 | 7.9±0.1 | 7.8±0.1 | |
| Turbidity (NTU) | 55.3±19.6 | 30.7±14.7 | 30.2±11.1 | 11.9±4.6 | |
| Salinity (%_o_) | 2.9±1.0 | 1.1±0.6 | 9.3±3.1 | 15.3±1.4 | |
| Dissolved oxygen (mg/L) | 7.4±0.6 | 4.5±0.2 | 4.5±0.6 | 4.8±0.4 | |
| Average water temperature (°C) | 26.1±0.1 | 27.2±0.2 | 28.9±0.3 | 29.2±0.3 | |
| Cumulative precipitation (mm) | 294 | 317 | 317 | 324 | |
| Average daily precipitation (mm) | 97.8 | 63.3 | 32.4 | 23.1 | |

Comment:

Discussion: Appropriate

Response:

We thank the reviewer for this affirmation.

Comment：

Bibliography: All references in text and bibliography except for those noted in table 2.

Response:

We have rechecked and updated the references lists in the bibliography.
